# Inhibition of MRN activity by a telomere protein motif

**DOI:** 10.1038/s41467-021-24047-2

**Published:** 2021-06-22

**Authors:** Freddy Khayat, Elda Cannavo, Majedh Alshmery, William R. Foster, Charly Chahwan, Martino Maddalena, Christopher Smith, Antony W. Oliver, Adam T. Watson, Antony M. Carr, Petr Cejka, Alessandro Bianchi

**Affiliations:** 1 Genome Damage and Stability Centre, School of Life Sciences, University of Sussex, Brighton, UK; 2 Institute for Research in Biomedicine, Faculty of Biomedical Sciences, Università della Svizzera italiana (USI), Bellinzona, Switzerland; 3 Department of Biology, Institute of Biochemistry, Eidgenössische Technische Hochschule (ETH), Zürich, Switzerland; 4 Present Address: SyntheX, Inc., San Francisco, CA USA; 5 Present Address: Department of Molecular Cell Biology, Weizmann Institute of Science, Rehovot, Israel

**Keywords:** Genomic instability, DNA damage and repair

## Abstract

The MRN complex (MRX in *Saccharomyces cerevisiae*, made of Mre11, Rad50 and Nbs1/Xrs2) initiates double-stranded DNA break repair and activates the Tel1/ATM kinase in the DNA damage response. Telomeres counter both outcomes at chromosome ends, partly by keeping MRN-ATM in check. We show that MRX is disabled by telomeric protein Rif2 through an N-terminal motif (MIN, MRN/X-inhibitory motif). MIN executes suppression of Tel1, DNA end-resection and non-homologous end joining by binding the Rad50 N-terminal region. Our data suggest that MIN promotes a transition within MRX that is not conductive for endonuclease activity, DNA-end tethering or Tel1 kinase activation, highlighting an Achilles’ heel in MRN, which we propose is also exploited by the *RIF2* paralog *ORC4* (Origin Recognition Complex 4) in *Kluyveromyces lactis* and the *Schizosaccharomyces pombe* telomeric factor Taz1, which is evolutionarily unrelated to Orc4/Rif2. This raises the possibility that analogous mechanisms might be deployed in other eukaryotes as well.

## Abstract (web summary)

Telomeres suppress the DNA damage response at chromosome ends. Here the authors show that in budding yeast the activity of the MRX complex in DNA repair and DNA damage signaling is inhibited by telomeric protein Rif2 via a short motif at the N-terminus.

## Introduction

Telomeres make a crucial contribution to genome stability by ensuring both the full replication of chromosome ends, through the action of the telomerase enzyme (reviewed in ref. ^1^), and their protection, through the agency of the telomeric complex (shelterin in vertebrates, reviewed in ref. ^2^). Telomerase synthesises short DNA repeats onto chromosome ends, which serve as a platform for the binding of telomere-specific and accessory factors. These factors, in turn, modulate the activity of the telomerase enzyme, and the feedback at each telomere determines the ultimate length of the telomeric DNA repeat array, which is mostly present in double-stranded form but terminates in a single-stranded 3′ overhang. Although it was early on predicted that telomeres would need to be equipped with mechanisms to disable the various arms of the DNA damage response (DDR) machinery, it was perhaps unanticipated that several of the DDR factors would be instrumental in ensuring telomere end processing and telomerase activity, as first documented in budding and fission yeast^3–6^. While several differences in the way the telomeric complex interacts with and affects DDR factors in different organisms are well documented, several common themes have emerged (reviewed in refs. ^7,8^). An example where elements of the DDR have been co-opted for telomere function is in the activation of telomerase. In the budding yeast *Saccharomyces cerevisiae*, the MRX complex, which is composed of Mre11, Rad50 and Xrs2 (Nbs1 in most other organisms) and is responsible for the recruitment/activation of the DDR kinase Tel1 (the ATM ortholog in yeast), promotes telomerase action: ablation of any of the three subunits, or of Tel1, leads to dramatic telomere shortening^3,9,10^. Similar requirements for the Tel1/ATM or the ATR (Rad3 in fission yeast) kinase for telomerase activation take place in mammalian cells and in fission yeast^11–13^.

The MRN complex is instrumental in orchestrating both DDR signalling and DNA repair, and acts as the main sensor of double-strand breaks (DSBs)^14^. The complex is equipped with nuclease and ATPase activity by Mre11 and Rad50 subunits, respectively, and has some structural similarity to the SMC family of proteins, with elongated coiled-coil (CC) motifs from the Rad50 subunits, which fold back onto themselves at a Zn hook formed at a CXXC motif found at the centre of the coiled region in Rad50. At the base of the CC region, a globular domain is formed where ATP-binding cassettes from two molecules of Rad50 come together and join an Mre11 dimer, in a tetrameric assembly. Nbs1/Xrs2 contains several protein interaction modules and is responsible for binding the Tel1/ATM kinase^15–17^. ATP binding and hydrolysis by Rad50 lead to conformational changes within the complex, and it has been proposed that the transition from an ATP-bound ‘closed’ complex to an ‘open’ one after ATP hydrolysis regulates the multiple activities of MRN^18–21^. The ATP-bound ‘closed’ complex is required for the tethering of DNA ends, which promotes one of the two main pathways for the repair of DSBs, non-homologous end joining (NHEJ)^20^. MRX/MRN stimulates end-joining both in budding and fission yeast^22,23^. The ATP binding and hydrolysis by Rad50 are also required to activate Tel1/ATM^24,25^. On the other hand, the ‘open’ state that results from ATP hydrolysis is competent for DNA binding by Mre11 and nucleolytic action, whereas Rad50 in the ATP-bound state blocks access to the nuclease-active sites^26,27^. The Sae2 factor is required for the endonuclease action of Mre11^28^. Endonucleolytic cleavage also appears to require ATP hydrolysis and not just release from the ‘closed’ state, so it has been proposed that an intermediate state between ‘closed’ and ‘open’, yet to be observed, might exist to promote endonucleolytic action^14^. The endonuclease action of Mre11 is followed by an exonucleolytic one to resect DNA and help channel DSB repair away from NHEJ and toward homology-dependent repair (HDR)^29,30^.

Telomeres must deal with the multiple threats posed by MRN in potentially initiating NHEJ, HDR, or DDR signalling. Mammalian telomeres engage the telomere-binding protein TRF2 to suppress ATM signalling, presumably via formation of t-loop structures, and to directly bind Nbs1 to modulate repair outcomes at telomeres^30,31^. In budding yeast, a different mechanism for MRX control has been described, which relies on telomere protein Rif2. Rif2 is unique to budding yeast and closely related species (see below) and interacts with the C-terminal domain (RCT) of the main DNA-binding protein of budding yeast telomeres, Rap1, for its association with telomeres, where it suppresses telomere elongation and NHEJ^32,33^. The first clues that Rif2 is linked to the regulation of MRX-Tel1 came from epistasis analysis of telomere length and from a screen for suppressors of hydroxyurea sensitivity in Tel1-overexpressing cells, which produced Rif2^34,35^. Rif2 inhibits MRX-dependent resection in G1^36^ and the tethering of DNA ends after DSB formation^23^. Interestingly, the binding of MRX to DSB is not severely diminished in the absence of Rif2, suggesting a regulatory role for Rif2^23,37^, an idea that is supported by the observation that Rif2 affects the ATPase activity of Rad50^23^.

We focus here on a conserved motif within Rif2, previously shown to be involved in the regulation of telomere length and Tel1 activation^38,39^, which we refer to as MIN for *M*RN-*in*hibitor. We show, by a combination of genetic and biochemical approaches, that the motif is a potent inactivator of MRX-Tel1, as it is capable of disabling the action of MRN-Tel1 in telomerase activation, NEHJ suppression and endonucleolytic digestion of DNA. Our data are consistent with a direct action of the motif on the N terminus of Rad50, and an effect on the ATP hydrolysis-dependent, Rad50-mediated allosteric transition enacted by Rad50 on the complex. We suggest that MIN provides a powerful and comprehensive mechanism for telomeres to inactivate the multiple actions of MRN-Tel1 at chromosome ends and that this mechanism has independently arisen in multiple lineages during fungal evolution.

## Results

### Identification of a conserved motif in Rif2 and Orc4

**Fig. 1:**
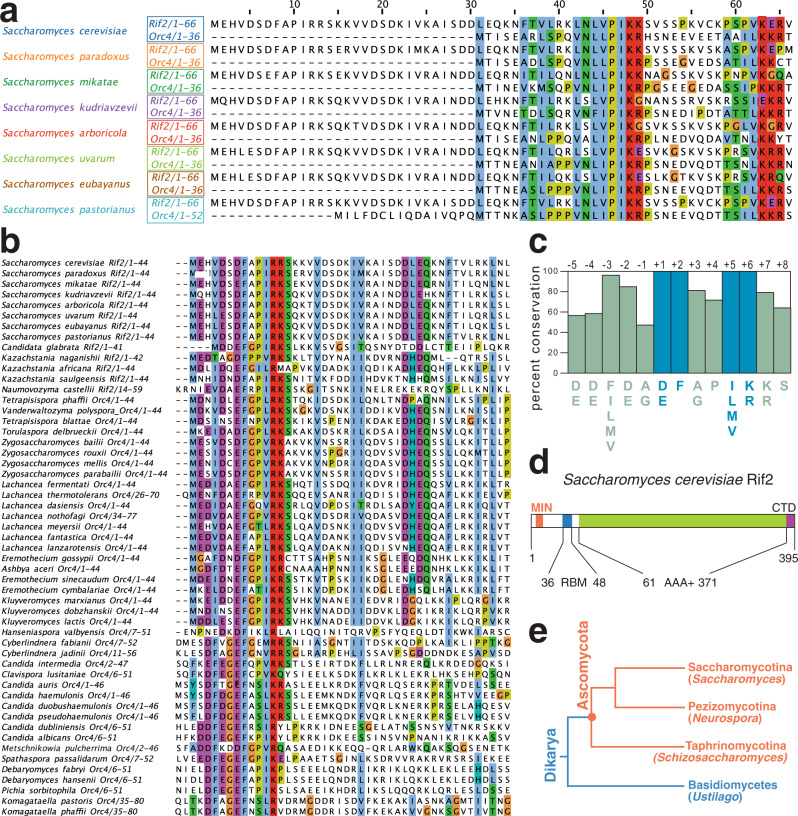
A conserved protein motif at the N terminus of Rif2 and Orc4 in the Saccharomycotina. **a** Sequence alignments of the indicated Rif2 and Orc4 proteins from Saccharomycotina species where Rif2 is present were produced in Clustal Omega and gaps were manually eliminated. Amino acid included are indicated after the protein name. Alignments were done using the Clustal Omega interface on the EBI site. Alignments were edited (in this case) and imaged in Jailview using Clustalx colouring of conserved residues (panels **a**, **b** and Fig. 7a). **b** Sequence analysis as in (**a**) of Rif2 and Orc4 N-terminal regions from species where the conserved region identified in the N-terminal extension in (**a**) is present. Species are broadly arranged top to bottom according to the phylogeny described in ref. ^74^. **c** Diagram illustrating the prevalence of conserved residues in the N-terminal cluster derived from the alignment in (**b**), defining the MIN motif. Based on the presence of four invariant residues (see also Fig. 7b) we adopt for the motif the numbering indicated on top of the diagram (invariant positions at +1, +2, +5 and +6, in blue). **d** Diagram of the domain structure of budding yeast Rif2. Conserved domains previously identified in ScRif2 include an AAA+ fold shared with Orc4, and a Rap1-binding motif (RBM) at the N terminus and one (CTD, C-terminal domain) at the C terminus^61^; the position of the MIN motif is shown in orange. **e** Phylogenetic tree, showing the relationship among the three clades comprising the Ascomycota. Representative species are indicated within parenthesis. Adapted from ref. ^75^.

*RIF2* evolved from the gene coding for the Origin Recognition Complex 4 subunit (ORC4) after a whole-genome duplication (WGD) event that occurred in budding yeast and related species (see below Fig. 1e for an illustration of evolutionary relationships of species discussed in this study)^33,40^. Therefore, to gain insight into the parts of Rif2 of possible functional significance (Fig. 1d), we decided to carry out an analysis of the Rif2 and Orc4 amino acid sequence aimed at identifying conserved and diverging regions. All yeast species bearing *RIF2* also carry a copy of *ORC4*, the latter being an essential gene. Clustal Omega alignment of Rif2 and Orc4 protein sequences from several fungal species in the Saccharomycetceae clade revealed that Rif2, compared to its Orc4 counterpart, consistently bore an extra stretch of ~30 amino acid at the N terminus (Fig. 1a). Strikingly, a survey of Orc4 sequences in other Saccharomycotina species devoid of Rif2 revealed that several Orc4 proteins share homology at their N terminus to the N-terminal ‘tail’ region in Rif2 (Fig. 1b). A strongly conserved consensus motif can be derived from this analysis and is summarised in Fig. 1c. Four positions, in particular, appear to be invariantly conserved and correspond to positions 7 (D/E), 8 (F), 11 (ILMV) and 12 (KR) of *S. cerevisiae* Rif2, and we propose that these residues (in blue in Fig. 1c) delimit a core motif of functional significance (see below). In this manuscript, we will refer to this motif as the MIN motif, for *M*RX-*in*hibitory motif, based on its proposed function.

### The MIN motif regulates telomere length through the MRX-Tel1 pathway

**Fig. 2:**
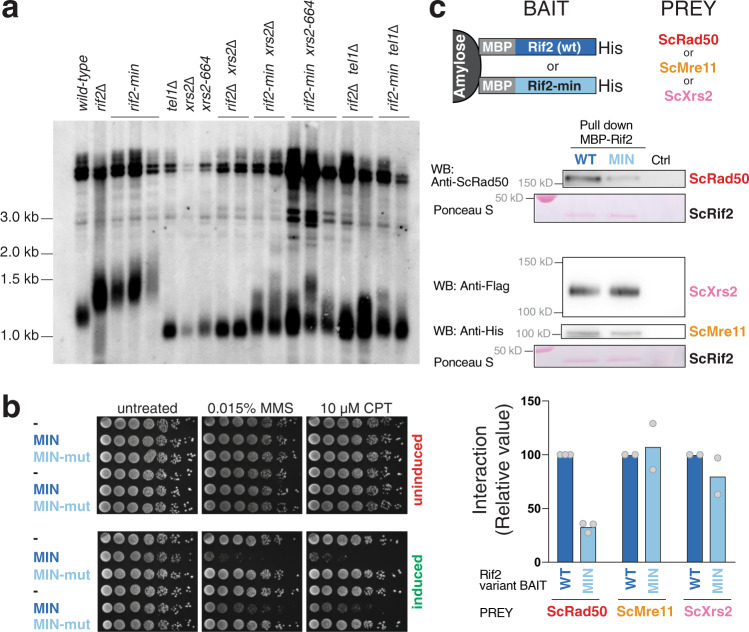
The MIN motif of Rif2 counteracts the action of the MRX complex in promoting telomere elongation and promotes binding to Rad50. **a** Southern blot analysis of telomere length of *Saccharomyces cerevisiae* strains of the indicated genotypes. Genomic DNA was digested with *Xho*I and detected using a telomeric probe. ‘Δ’ indicates null alleles obtained by full deletion of the coding sequence. The *rif2-min* allele carries an F8E P10N substitution. **b** Viability assays of strains overexpressing a fusion protein containing the first 34 amino acids of Rif2 under control of the galactose-inducible promoter, either in wild-type form (MIN) or mutant (MINmut, carrying the double F8E P10N substitution). Strains were grown in glucose-containing rich medium (YPAD) before plating onto either glucose (uninduced, top row) or induced (galactose, bottom row) plates containing the indicated chemicals and incubated at 30 °C for 2–3 days. **c** Protein interaction assays with immobilised wild-type or mutant Rif2 (Rif2-min) as bait, and Rad50, Mre11 or Xrs2 proteins used individually as prey. The bound proteins were separated by electrophoresis and detected by Western blotting (for Rad50, anti-Rad50; for Mre11-his, anti-his; and for Xrs2-FLAG, anti-FLAG), or by Ponceau S staining (for Rif2 wild-type or Rif2-min). CTRL lane is prey protein incubated with amylose resin without bait. A representative blot is shown, as well as a quantification of 2–3 independent ones, as indicated. Source data are provided as a Source Data file.

Previous scanning mutagenesis analysis of *RIF2* revealed that a small N-terminal region in Rif2 is responsible for regulating telomere length in budding yeast and defined the first 60 amino acids of ScRif2 as the BAT domain, for ‘Blocks Addition of Telomeres’^38^. The BAT domain fully contains the MIN motif and the mutagenesis investigation of BAT is broadly consistent with the analysis of sequence conservation (Fig. 1b), in that some of the most strongly conserved positions within the Sc MIN (particularly F8) were found to have the strongest telomere length phenotype when changed to alanine^38^. Intriguingly, BAT lays within a small N-terminal fragment previously proposed to interact with the C terminus of Xrs2 and to compete for its binding to Tel1^37^. This raised the possibility that MIN might function by promoting the binding of Rif2 to Xrs2 and thus disabling its critical function in the Tel1 activation of telomerase. Although earlier experiments concluded that BAT does not act by affecting the function of the Xrs2 C terminus in telomere length regulation^38^, this analysis was carried out with an *xrs2* allele only partly defective for Tel1 binding, and showing only a mild telomere shortening phenotype^41^. In fact, Xrs2 carries two putative Tel1-interacting motifs at its C terminus and impairment of both motifs is required to confer significant telomere shortening^16,38,42^. In order to determine whether the Rif2 MIN motif acts through the MRX-Tel1 pathway of telomerase regulation, we carried out epistasis analysis of telomere length using *xrs2*- and *tel1*-null alleles, as well as C-terminal truncation of Xrs2, which ablates both Tel1-interacting motifs in the protein and fully disables Tel1 recruitment to telomeres^42,43^. This allele, *xrs2-664*, confers telomeres as short as the *xrs2*- or *tel1*-null alleles and is, therefore, an ideal choice for epistasis analysis (Fig. 2a). As expected, a *rif2* allele carrying two-point mutations within the MIN motif (F8E and P10N mutations, *rif2-min* allele) generated telomeres at least as long as the *rif2* null. An examination of telomere length in the double mutants carrying the null allele pairings *rif2-Δ xrs2-Δ* and *rif2-Δ tel1-Δ* showed, as anticipated based on the previously recognised role of Rif2 in modulating MRX-Tel1 function^35,44^, a dramatic shortening of telomere length near the levels seen in the *xrs2-Δ* and *tel1-Δ* single mutant strains. Importantly, we observed similarly shortened telomeres in double mutant configurations where the *rif2*-*Δ allele* was replaced by the *rif2-min* allele, suggesting that the Xrs2 C terminus and the Tel1 kinase are both required for the telomere elongation effect conferred by loss of the MIN motif in Rif2. Thus, as mutations in the MIN motif are epistatic to *xrs2/tel1*, the simplest interpretation of these results is that MIN acts by impairing the pathway of telomerase activation that relies on the MRX-dependent recruitment of the Tel1 kinase.

If the MIN motif impairs the action of the MRX-Tel1 complex, it is possible that ectopic overexpression of only the N-terminal Rif2 region containing MIN, incapable to locate telomeres due to the lack of binding to Rap1, might render cells hypersensitive to genotoxic stress, reminiscent of MRX mutants. In order to test this idea, we over-expressed peptides bearing the first 34 amino acid residues of Rif2 from the strong galactose-inducible promoter. In line with the hypothesis above, we found that overexpression of the MIN motif, but not a mutant version bearing the F8E and P10N mutations, rendered the cells hypersensitive to treatment with methyl methanesulfonate (MMS) or camptothecin (CPT) (Fig. 2b). Thus, expression of this large truncation allele of Rif2, containing only the MIN motif, has a dominant-negative effect on the ability of cells to enforce DDR, consistent with a possible inactivating role on MRX.

Tel1 is pivotal in the telomerase-dependent pathway of telomere maintenance in budding yeast, but less crucial in assisting the DNA repair function of the MRX complex in budding yeast^23,45^. In particular, Tel1 is required to guarantee resistance to genotoxic stress by CPT, but not MMS^46^. If the MIN motif acted solely by affecting Tel1 recruitment to the complex, via the Xrs2 C terminus, its overexpression might have been expected to have displayed specificity towards CPT sensitivity, instead of affecting sensitivity to MMS as well, as we observed (Fig. 2b). We, therefore, decided to address whether Rif2 might function by targeting other subunits of the MRX complex instead of the Xrs2 C terminus. In pull-down assays with purified components, Rif2 was found to be able to interact with all three subunits of the complex (Fig. 2c). Interestingly, the F8E P10N mutations reduced, although they did not abolish, the ability of Rif2 to interact with Rad50, but not with Mre11 and Xrs2, suggesting that the interaction with Rad50 might be the more relevant one with regard to the action of the MIN motif. Taken together, these results indicate that the MIN motif in Rif2 acts at telomeres by directly affecting the action of the MRX complex. In agreement with a recent biochemical study^39^, this effect of MIN is likely exerted through an interaction on the Rad50 subunit. While that study has reported that an F8A mutation in Rif2, which impairs the ability of Rif2 to modulate MRX, does not affect the interaction with Rad50, the double mutant studied here shows diminished binding, suggesting that multiple contributions are made by MIN motif residues in contacting Rad50^39^.

### The MIN motif is required for the suppression of checkpoint activation at telomeres by Rif2

**Fig. 3:**
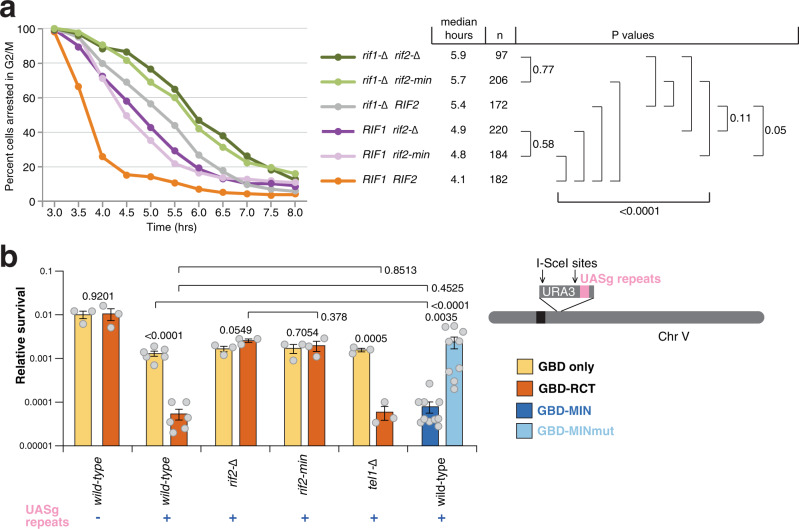
The MIN motif enforces the suppression of the DDR and NHEJ at a DSB flanked by telomeric repeats. **a** A specific DSB, flanked by telomeric repeat arrays at both ends, was induced at the ADH4 locus on the left arm of chromosome VII by expression of the HO endonuclease in galactose-containing medium. After induction of the break, cells in the S phase were monitored for their ability to escape G2/M arrest. The percentage of G2/M-arrested cells, from at least three independent experiments for each genotype (two for rif1-Δ), was estimated using the Kaplan–Meier analysis by GraphPad Prism (log-rank test). **b** Viability assays to quantify the ability of cells to survive a double-inducible DSB on either side of the URA3 gene on chromosome V. Strains were grown in minimal medium lacking uracil and tryptophan, and then plated out on plates containing glucose (to assess plating efficiency) or galactose (to assess survival after cleavage). GBD is Gal4 DNA-binding domain, RCT is the Rap1 C-terminal domain, MIN contains Rif2 amino acids 1–40, and the MINmut version carries the F8E P10N mutations. Relative survival was determined from at least three independent experiments; the number of independent cultures analysed was 3, 3, 6, 6, 3, 3, 3, 3, 3, 3, 10, 9 for strains from left to right. Data are presented as mean with SEM. *P* values are reported for each pair of datasets as indicated and were obtained with a two-tailed, unpaired *t* test. Source data are provided as a Source Data file.

Rif2 has a protective function at telomeres by both preventing exonucleolytic degradation and activation of the DDR^47,48^. Because the MRX-Tel1 complex contributes to the DDR response at unprotected telomeres, we tested the hypothesis that Rif2 might carry out its role in suppressing DDR activation at telomeres through the action of the MIN motif. To do so, we employed an assay first described by the Weinert lab that relies on the induction of a DSB by the HO endonuclease at a chromosomal site flanked by telomeric repeats^49^. In our setting, the DSB is flanked at both ends by telomeric repeats^48^. The DNA break was induced, and small-budded cells, indicative of the early S-phase stage, were micro-manipulated on a plate, and then inspected over several hours to measure the kinetics of progression through the cell cycle. Specifically, the length of the arrest in the G2/M phase following the break, as indicated by dumbbell-shaped cells, was monitored: while wild-type cells are expected to cycle quickly, as the DDR is not activated due to the protective effect of the telomeric repeats flanking the newly generated DNA ends, mutations affecting telomere protection will disable the checkpoint-suppressive function and result in transient cell cycle arrest in G2/M. In this assay, checkpoint activation is affected by Mre11, and increases in resection and in Mre11 and Tel1 association are observed at the break^48^. At least two independent pathways contribute to checkpoint suppression in this setting, one dependent on Rif1 and one dependent on Rif2^48^. We set out to test whether the contribution of Rif2 to checkpoint suppression relies on the MIN motif. As previously described, loss of Rif1 and Rif2 caused additive delays in the release from G2/M in this assay (Fig. 3a). Remarkably, in both the presence or absence of Rif1, the *rif2-min* allele produced the same effect as the *rif2*-null allele (Fig. 3a), indicating that the MIN motif is required for the ability of Rif2 to prevent checkpoint activation at unprotected telomeric DNA ends.

### The MIN motif of Rif2 suppresses NHEJ

In budding yeast, the MRX complex is required to promote DSB repair by NHEJ, and Rif2 suppresses NHEJ at telomeres^33^. Because we showed that the Rif2 MIN motif limits the ability of MRX to promote telomere length, we addressed whether the motif might also be responsible for the disabling effect of Rif2 on NHEJ. DSB repair events can be channelled into the NHEJ pathway in the absence of an intact homologous sequence that could serve as a template for repair by homologous recombination. NHEJ can be measured by assessing the ability of haploid budding yeast cells to survive a double DSB flanking a selectable marker^33^. In this setting, telomeric sequences positioned near one of the two breaks cause decreased survival upon DSB induction, due to their suppression of NHEJ, at least partly via the Rap1-promoted recruitment of Rif2^33^. The effect of telomeric repeats in this assay can be bypassed by directly tethering the RCT near the DSB by fusing it to the Gal4 DNA-binding domain (GBD) and placing copies of its recognition site (UASg) near the break. As expected, the presence of UASg sites near the break led to decreased survival upon the presence of a GBD-RCT fusion protein (Fig. 3b). Deletion of Rif2 rescued the viability defect, due to loss of NHEJ inhibition and consequent DSB healing. Crucially, the effect of the UASg/GBD-RCT combination depended on the presence of a functional MIN motif in Rif2, as the phenotypes of *rif2-Δ* and *rif2-min* strains were indistinguishable in this assay, indicating that the motif is required for the suppression of NHEJ by Rif2.

The observation that the role of the BAT domain in telomere length suppression did not require the RCT domain of Rap1^38^ predicts that tethering MIN directly to the break site, via the expression of a GBD-MIN fusion, should mimic the effect seen with the tethered RCT domain. Indeed, a decrease in survival was obtained when the first 40 amino acids of Rif2 were fused to GBD, while the F8E P10N double mutation abolished the NHEJ-suppressive effect of this small Rif2 N-terminal fragment fusion (Fig. 3b). Taken together, these results indicate that the MIN motif of Rif2 is both necessary and sufficient to achieve the suppression of NHEJ in DSB repair by Rif2.

Interestingly, Tel1 was not required for the RCT to suppress NHEJ, suggesting that the effect of MIN in this setting occurs via the MRX complex itself, rather than via targeting the action of the Tel1 kinase, unlike in the case of the regulation of telomere length. This result is consistent with the suggestion, as described above, that the MIN motif acts via an interaction with Rad50, rather than by affecting the ability of Xrs2 to recruit Tel1, as originally proposed for Rif2^37^. Because the *rif2-min* allele mimics the *rif2* null with regard to both telomere length and telomere protection, we think it is unlikely that the MIN motif, and by extension Rif2, acts via the Xrs2-Tel1 recruitment axis, even in telomere length regulation. Rather, these results are consistent with a model recently proposed whereby the Rif2 N-terminal region affects the ability of the MRX complex to activate the Tel1 kinase and specifically the ability of the Rad50 subunit to trigger its activation^25,39^. Our results indicate that MIN affects a broad spectrum of MRX activities besides Tel1 activation, including specifically NHEJ.

### The MIN motif disables the endonucleolytic function of MRX

**Fig. 4:**
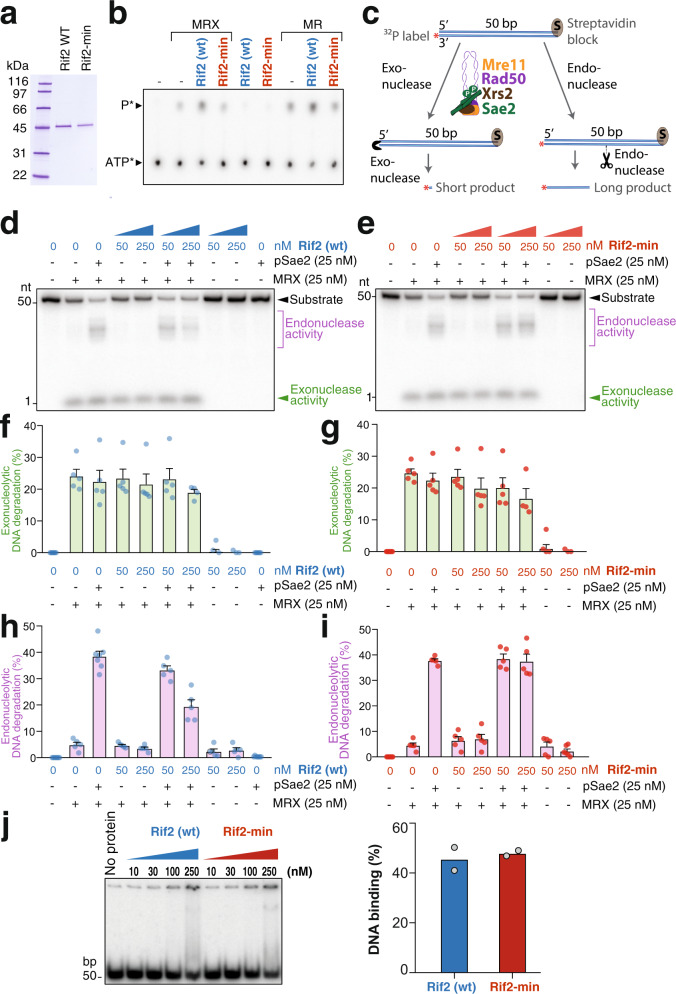
The MIN motif of Rif2 is required to suppress the endonucleolytic activity of MRX. **a** Purified *S. cerevisiae* wild-type Rif2 and MIN-mutated Rif2-min variants used in this study. The polyacrylamide gel was stained with Coomassie Blue. **b** Representative ATPase assay with MRX (100 nM), MR (100 nM), wild-type Rif2 (500 nM), or Rif2-min (500 nM), as indicated. **c** A scheme of the nuclease assay used to monitor the exonuclease and endonuclease activities of the MRX complex in the presence of phosphorylated Sae2. The radioactive label is at the 3′ position on the bottom DNA strand. The 3′–5′ exonuclease activity gives rise to a small product that migrates at the bottom of the gel. The endonuclease activity yields longer products. **d** Nuclease assays with MRX, phosphorylated Sae2 (pSae2) and wild-type Rif2, as indicated. Shown is a representative experiment. **e** Nuclease assays as in (**d**), but with Rif2-min (F8E P10N mutations). **f**, **g** Quantitation of exonucleolytic data such as shown directly above in (**d**, **e**), respectively. Error bars represent SEM for five independent experiments. **h**, **i** Quantitation of endonucleolytic data as shown directly above in (**d**, **e**), respectively. Error bars represent SEM for five independent experiments. **j**, Left panel: representative electrophoretic mobility shift assays with Rif2 (wt) and Rif2-min with the indicated protein amounts. Right panel: quantitation of data such as in the gel shown at left, with 250 nM Rif2 (wt) or 250 nM Rif2-min; *n* = 2, averages and individual data points are shown. Source data are provided as a Source Data file.

Our in vivo data indicate that the functions of Rif2 in telomere length regulation, suppression of the DNA damage checkpoint at telomeres, and inhibition of NHEJ at telomeres are mediated by the MIN motif. In addition, epistasis analysis implicated the MIN motif of Rif2 in the control of MRX-Tel1, and mutating the motif diminished its ability to physically interact with Rad50, firmly linking the MIN motif with MRX functionality. To get insights into the mechanism of action of the MIN motif on the MRX complex, we expressed and purified recombinant full-length Rif2, in wild-type and the F8E P10N mutant form (Fig. 4a), and tested its effect on various activities of the purified MRX complex. As observed previously^23^, Rif2 was found to stimulate the ATPase activity of the MRX and MR complexes (Fig. 4b). Importantly, the F8E P10N mutations ablated this stimulatory effect (Fig. 4b). The ATPase activity of the Rad50 subunit is instrumental in promoting conformational changes in the complex, which modulate its activity in DNA end resection and tethering as well as Tel1 kinase activation^20^. This raised the possibility that the MIN motif of Rif2 might directly impair the nucleolytic function of the MRX complex. To test this, we assayed nuclease activities of the MRX complex in the presence of wild-type and mutant Rif2. Previously, it has been established that MRX has a 3′–5′ exonuclease activity that does not require ATP hydrolysis (Fig. 4c, left), and Mre11 alone is necessary and sufficient for this function^50,51^. In addition, MRX employs its endonucleolytic activity to resect DSBs with protein blocks. The endonucleolytic cleavage by the nuclease of Mre11 requires ATP hydrolysis by Rad50, as well as phosphorylated Sae2 (pSae2) as a co-factor (Fig. 4c, right)^28,52^. We employed a substrate used previously, which allows monitoring both exonuclease and endonuclease activities simultaneously (Fig. 4c)^52^. We observed that Rif2, whether in its wild-type or mutant form, had no effect on the exonuclease action of MRX, while wild-type Rif2, but not the Rif2-min mutant, was found to strongly reduce the endonuclease action of MRX in the presence of Sae2 (Fig. 4d–i). This inability of the mutant to suppress endonucleolytic cleavage was not due to decreased DNA-binding activity, as both mutant and wild-type variants of Rif2 retained DNA-binding capability (Fig. 4j).

Rad50 is a key regulatory component in the MRX complex, which triggers ATP-dependent conformational switches to regulate its various functions. ATP binding by Rad50 is required for Tel1 activation by MRX and end tethering in NHEJ^19,20,53^, and ATP hydrolysis is required for the Rad50-dependent endonucleolytic action of Mre11^28,50^. Accordingly, Rad50 mutants lacking ATP-binding activity recapitulate the multiple phenotypes of the null allele^54^. The ability of Rif2 to interact with Rad50, which is at least partly dependent on the MIN motif, and the requirement of the MIN motif to stimulate the Rad50 ATPase activity, suggest that Rif2 inactivates the multiple functions of MRX by triggering non-productive ATP hydrolysis by the MRX complex, and hence enforcing ATP-dependent conformational changes of the complex. It was recently shown that the F8 residue on Rif2 is instrumental in impairing the ability of Rad50 to activate Tel1, explaining the telomere length phenotype of the MIN motif mutants, which we here show is epistatic to the Xrs2-Tel1 pathway (Fig. 2)^39^. The suppression of NHEJ that we now report is also consistent with a MIN-dependent switch of the complex to an ‘open’ conformation after ATP hydrolysis. The nuclease data presented here further support this model, as we show that only the ATPase-dependent endonuclease activity of MR (or MRX) is affected, but not the ATPase-independent exonuclease activity. In this scenario, MIN is proposed to promote ATP hydrolysis by MRX (or MR) in a non-productive manner and thus prevent productive hydrolysis that would lead to endonucleolytic action. This conclusion agrees with previous data suggesting that Rif2 discharges the ATP-bound form of Rad50 to attenuate Tel1 activity^39^. In summary, these results support a scenario where the MIN motif of Rif2 converts the MRX-ATP complex to the open form, which broadly impairs the MRX complex in its function to promote DNA repair, DDR signalling and telomerase regulation.

### The MIN motif of Rif2 interacts with the N-terminal region of Rad50

Among the characterized alleles of the MRX subunits, the *rad50S* allele stands out in that it leads to telomere elongation, rather than shortening as is seen in *rad50*-null cells^10^. The *rad50S* alleles (generally represented by the *rad50-K81I* mutation) comprise a small group of mutations originally identified for their meiotic phenotype in being defective in the resection of meiotic DSBs and removal of Spo11 from the breaks^28,55^. The phenotype of *rad50S* mutants is similar in many respects to that of strains defective in the nuclease activity of Mre11 or lacking Sae2, which is required to stimulate the endonucleolytic action of Mre11 (reviewed in ref. ^56^). *Rad50S* cells, however, differ in one significant aspect, telomere elongation, as *mre11* strains defective in endonucleolytic activity have normal telomere length, as do *sae2*-null mutants^57,58^. The failure to promote nucleolytic processing of DNA ends, therefore, cannot explain the elongated telomeres promoted by Rad50S.

**Fig. 5:**
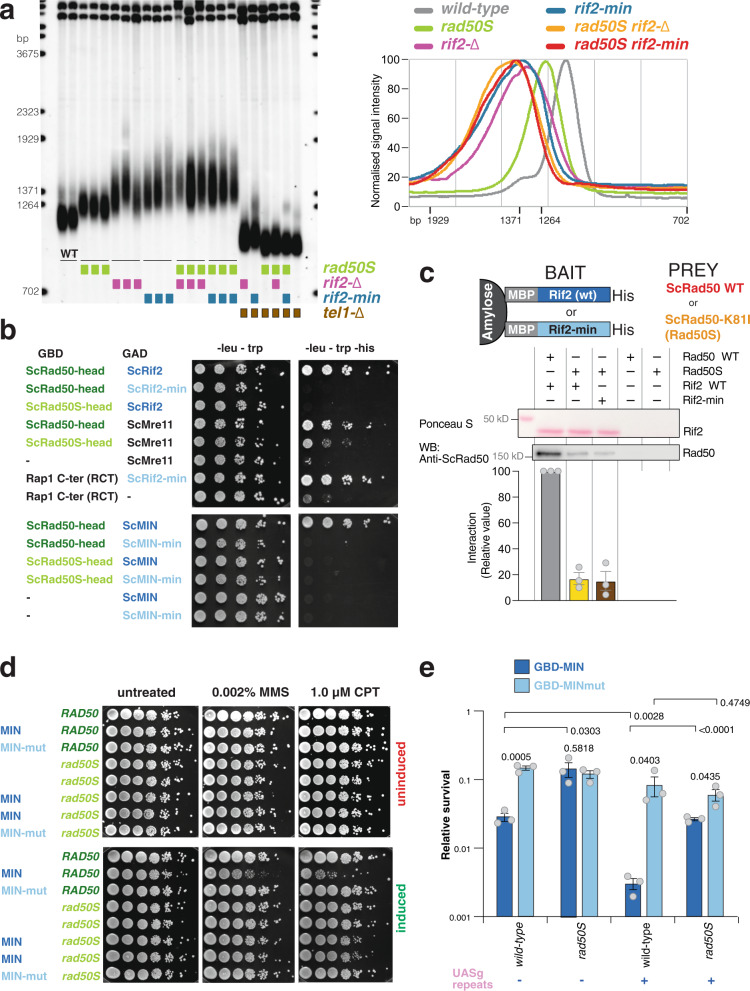
Rad50S (Rad50-K81I) fails to interact with the MIN motif of Rif2. **a** Southern blot analysis of telomere length of *Saccharomyces cerevisiae* strains of the indicated genotypes, carried out as described in Fig. 2a. A quantification of hybridization signals from this gel obtained with Image Gauge v4.1 (Fuji) is shown on the right panel; strains of identical genotypes were averaged together. **b** Yeast two-hybrid assays of the *S. cerevisiae* fusion proteins indicated. Plates are minimal medium lacking the nutrients indicated on top. Five-fold serial dilutions were spotted left to right and incubated for 3–4 days at 30 °C. **c** Protein interaction assays with immobilized wild-type or mutant Rif2 (Rif2-min) as bait, and Rad50 or Rad50-K81I (Rad50S) proteins as prey. The bound proteins were separated by electrophoresis and detected by Western blotting (with anti-Rad50 antibody) or by Ponceau S staining (for Rif2 wild-type or Rif2-min). A representative blot is shown, as well as a quantification of three independent ones, as indicated. Data are presented as mean with SEM. **d** Viability assays of strains overexpressing a fusion protein containing the first 34 amino acids of Rif2 under control of the galactose-inducible promoter, either in wild-type form (MIN) or mutant (MINmut), as in Fig. 2b. Uninduced strains were plated onto either glucose (uninduced, top row) or induced (galactose, bottom row) medium containing the indicated chemicals and incubated at 30 °C for 2–3 days. **e** Viability assays to quantify the ability of cells to survive a double-inducible DSB on either side of the URA3 gene on chromosome V were carried out as in Fig. 3b. GBD is Gal4 DNA-binding domain, MIN contains Rif2 amino acids 1–40 and the MINmut version carries the F8E P10N mutations. Relative survival was determined for three independent strains; error bars represent SEM. *P* values are reported for each pair of datasets as indicated and were obtained with a two-tailed, unpaired *t* test. Source data are provided as a Source Data file.

The *rad50S* mutations are clustered in a small region at the N terminus of the Rad50 protein, near the Walker A-type ATPase motif, in the part of the protein that contributes to half of the globular ‘head’ domain, and have recently been shown to define an area that is likely to provide the binding interface for phosphorylated Sae2, as the latter was shown to be incapable of binding the Rad50-K81I protein^52^. Prompted by the observation that the Rif2 MIN motif binds Rad50, we reasoned that the *rad50S* telomere phenotype could be explained if *rad50S* alleles failed to bind a repressor of MRX activity. We thus set out to test the idea that Rad50-K81I (from here onwards referred to as ‘Rad50S’) might be defective in MIN binding. To begin with, if the K81I mutation abolished the interaction with Rif2, then *rad50S* and *rif2-min* (or *rif2-Δ*) alleles would be predicted to be epistatic with regard to their effect on telomere length. As expected, single mutants showed telomere elongation, with *rif2* mutations conferring longer telomeres than *rad50S*, and *rif2-min* strains bearing slightly longer telomeres than *rif2*-null ones (Fig. 5a). Importantly, both *rad50S rif2-Δ* and *rad50S rif2-min* double mutant strains had telomeres of very similar length to *rif2* single mutants, indicating that these *rad50* and *rif2* alleles are largely epistatic, and consistent with the idea that an interaction between the two proteins is compromised in both mutants.

To test this idea directly, we performed yeast two-hybrid assays between wild-type and mutant variants of Rad50 and Rif2. Since we were interested to test interactions in the globular region of Rad50, we constructed a variant of the gene lacking the large central portion that codes for the CCs. As anticipated, we found that this variant, which we call Rad50head, is capable of interacting with Mre11 and, confirming the pull-down results with purified proteins (Fig. 2c), also with full-length wild-type Rif2 (Fig. 5b). Instead, the presence of either the *min* mutation in Rif2 (full-length and truncated) or the *S* mutation in Rad50 abolished the interaction in this assay. In agreement with this result, we also found that mutant Rad50S was impaired in its interaction with Rif2 in the pull-down assay with purified proteins (Fig. 5c). We note that the yeast two-hybrid assay appears to be more stringent as the interaction was not completely abolished in the pull-downs, no matter whether Rif2 or Rad50 was mutated (Figs. 2c and 5c), leaving open the possibility that other regions of Rad50 might interact with Rif2 (multiple interactions for both Rif2 and Sae2 are observed with various components of MRX; Fig. 2c^52^). However, we did not detect additive effects when testing the two mutant variants in this assay (Fig. 5c), suggesting that the specific interaction at this interface might be equally, and in terms of physiological relevance possibly completely, disrupted in the two mutants.

The interaction data support the epistasis analysis of telomere length in the mutants. To further evaluate the functionality of this interaction in vivo, we decided to test the possible suppressive effect of the *rad50S* allele on two of the MIN phenotypes that we described earlier. The interpretation of the increased sensitivity of cells overexpressing MIN to genotoxic agents (Fig. 2b) is that MIN directly interacts with MRX to disable its function in repair. So, if MIN requires the K81 residue to interact with Rad50, then the *rad50S* allele would be predicted to suppress the decreased viability for strains overexpressing MIN upon exposure to CPT and MMS. Indeed, in a *rad50S* background, we observed a rescue of the MIN-dependent toxicity to both these drugs (Fig. 5d). Similarly, we found that the ability of MIN to impair healing of a DSB by NHEJ when tethered near the break (Fig. 3b) was partly compromised in strains bearing the *rad50S* allele (Fig. 5e); in other words, *rad50S* suppressed the ability of MIN to inhibit NHEJ when tethered near the DSB. Interestingly, and in agreement with the MMS and CPT sensitivity data reported in Fig. 2b, MIN was still able to inhibit healing of the break even in the absence of tethering, suggesting that overexpression of a functional MIN has an overall dampening effect on NHEJ within the cell. Taken together, these results identify the region in Rad50 that is responsible for a functional interaction with Rif2 via the MIN motif. Further studies will be required to elucidate the interplay between Rif2, Sae2, and Rad50 interactions, but these data provide a molecular rationale for the longstanding observation of elongated telomeres in *rad50S* cells. The reason for the particular length setting in these cells, which is not as high as that seen in *rif2* mutants, is unclear. One possibility is that, although Tel1 activation after genotoxic stress in *rad50S* appears to be increased, the mutation might intrinsically impair full activation of Tel1 in the mutant complex even in the absence of Rif2^59^; alternatively, the Rad50/Rif2 interaction might be more weakly affected in vivo by the *rad50S* mutation compared to the ones in MIN, although this is not supported by our biochemical data.

### A role for the MIN motif at telomeres beyond Rif2

**Fig. 6:**
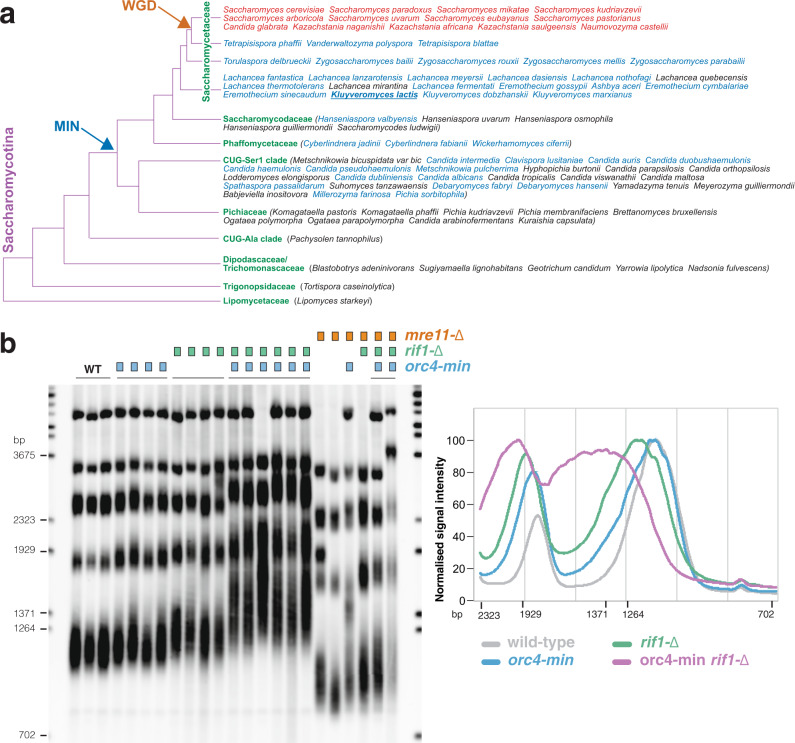
The MIN motif of *K. lactis* Orc4 suppresses telomere elongation. **a** Phylogenetic tree of the Saccharomycotina, adapted from ref. ^74^. Only species for which a search for Rif2 and/Orc4 was carried out are included. Species bearing Rif2 are in red; in all cases, Rif2 has MIN domain, whereas Orc4 does not. Species bearing putative MIN domain in Orc4 are in blue, whereas those where MIN domain is not present in Orc4 are in black. All red- and blue-lettered species have their MIN sequenced reported in Fig. 1b. The occurrence of the whole-genome duplication (WGD) event during the evolution of the Saccharomycetaceae clade is indicated by the orange arrow, whereas the deduced appearance of the MIN motif in Orc4 within the Saccharomycotina is indicated by the blue arrow. *Kluyveromyces lactis* is underlined. **b** Southern blotting of *Eco*RI-digested DNA hybridised with a telomeric oligonucleotide for the indicated genotypes. A quantification of hybridization signals from this gel obtained with Image Gauge v4.1 (Fuji) is shown on the right panel; strains of identical genotypes were averaged together. Source data are provided as a Source Data file.

Remarkably, as described above for the *Saccharomyces* genus (Fig. 1a), all the yeast species analysed, which bear both Rif2 and Orc4, display the motif in Rif2, but invariably lack it in Orc4 (Fig. 6a)^38,39^. These species belong to a clade within the Saccharomycetaceae that originated after the WGD event (species in red in Fig. 6a). Instead, almost every species within the Saccharomycetaceae, which lacked Rif2, displayed the MIN-containing N-terminal region Orc4 (Fig. 6a, species in blue), with only two exceptions (species in black). A group of species within the post-WGD clade are devoid of Rif2, presumably because the duplicated *ORC4* gene was lost early before it could differentiate into *RIF2*: these species also presented the MIN motif in Orc4. The analysis suggests that the MIN motif originated in Orc4 and that its function was then retained in Rif2 and lost from Orc4 (presumably because redundant) in the budding yeast *S. cerevisiae* and closely related species.

The proposition that the MIN motif has originated in Orc4 coupled with the suggestion that MIN in Rif2 acts to regulate the action of MRX-Tel1 in budding yeast raised the possibility that Orc4 might have originally had a similar role in affecting MRX-Tel1 before this function was taken up by Rif2. In order to test this idea, we turned to *Kluyveromyces lactis*, since this yeast (underlined in Fig. 6a) does not bear Rif2 and has a MIN-carrying Orc4. Similarly to budding yeast, MRX in *K. lactis* is required to maintain telomere length^60^, and mutations in the MIN motif of KlOrc4 would thus be predicted to lead to telomere elongation. We introduced the F8E and P10N mutations into KlOrc4 (*orc4-min*) and measured telomere length in the resulting strains. Although some variation was observed, a moderate level of telomere elongation was observed in *orc4-min* isolates, as longer telomeres were over-represented in these strains compared to wild type (Fig. 6b). Because the *Kl orc4-min* and *Sc rif2-Δ* telomere length phenotypes are relatively minor in comparison to that enforced by the lack of Rif1 in either species (Fig. 6b)^32^, to further confirm the elongation phenotype of the *orc4-min* allele, we combined it with a null mutation in *rif1*, since in budding yeast disabling both the Rif1- and Rif2-dependent pathways of telomere elongation has a synergistic effect^32^. Indeed, combining the *rif1-Δ* and *orc4-min* mutations led to a clear additive effect on telomere elongation, indicating that Rif1 and Orc4 independently regulate telomere length in *K. lactis*, the latter protein doing so through its MIN motif. We next combined the *orc4* and *rif1* mutations with a *mre11* deletion allele (Fig. 6b). Telomeres in the double and triple mutants were drastically shortened, indicating that *MRE11* is largely epistatic to *ORC4* and *RIF1* in telomere length regulation in *K. lactis*, consistent with the idea that Orc4 MIN acts by affecting MRX-Tel1. These results suggest an unanticipated role for Orc4 at telomeres and are consistent with subfunctionalisation of the Orc4 and Rif2 proteins after the WGD event, where Orc4 has retained a function in DNA replication, while Rif2 has taken up the role in MRX-Tel1 control at telomeres. It remains to be established whether Orc4 has a role in *K. lactis* telomeres as part of the ORC complex or whether it acts independently of it. As Rif2 is delivered to telomeres via the RCT of Rap1, one possibility is that Orc4 might also be recruited via an interaction with Rap1. Residues that are important in Rif2 for interaction with the RCT^61^ do not seem to be particularly well conserved in Orc4, and a bias towards conservation in the MIN-bearing Orc4 proteins is not apparent. Furthermore, some species that have an Orc4 MIN do not have a Rap1 RCT (e.g., *Candida albicans*). It remains therefore unclear whether the RCT of Rap1 might play a role in recruiting Orc4 at telomeres in some settings^62^, but we note that the role of human telomeric protein TRF2 in recruiting ORC to telomeres has been reported^63^. Both the dynamics and mechanism of telomere recruitment of Orc4 to fungal species will require further investigation.

In seeking to understand how general a role MIN might play in the regulation of the MRX complex, we sought to determine how widespread MIN is in ORC4 genes. Within the Saccharomycotina, the MIN motif was found to be present in the Phaffomycetaceae and CUG-Ser-1 clades, and possibly in one species within the Saccharomycodaceae (Fig. 6a, species in blue). In the Ascomycota, we failed to identify the MIN motif in any of the remaining groups within the Saccharomycotina (see four bottom clades in Fig. 6a), as well as in the two other groups, which together with the Saccharomycotina comprise this group (the Pezizomycotina—four species examined—and the Taphrinomycotina—12 species); we also could not find it in the Basidiomycota (three species) (see Fig. 1e). It therefore appears likely that the MIN motif evolved in Orc4 at some point within the Saccharomycotina, and that several species within this group lost it in Orc4 afterwards. Although we conducted searches for the motif in the genomes of some of the candidates where it would be most strongly predicted to be present, due to their belonging to clades where it is prevalent (e.g., *Lachancea quebecensis* and *mirantina*, and *Candida tropicalis*), we failed to find clear hits. It is unclear how the function of MIN is fulfilled at the telomeres of species that are devoid of it, but these results are consistent with the emerging remarkable modularity and flexibility of the telomeric complex in dealing with its multiple tasks (within shelterin, e.g., several differences have been documented between the human and mouse complex, reviewed in ref. ^7^).

**Fig. 7:**
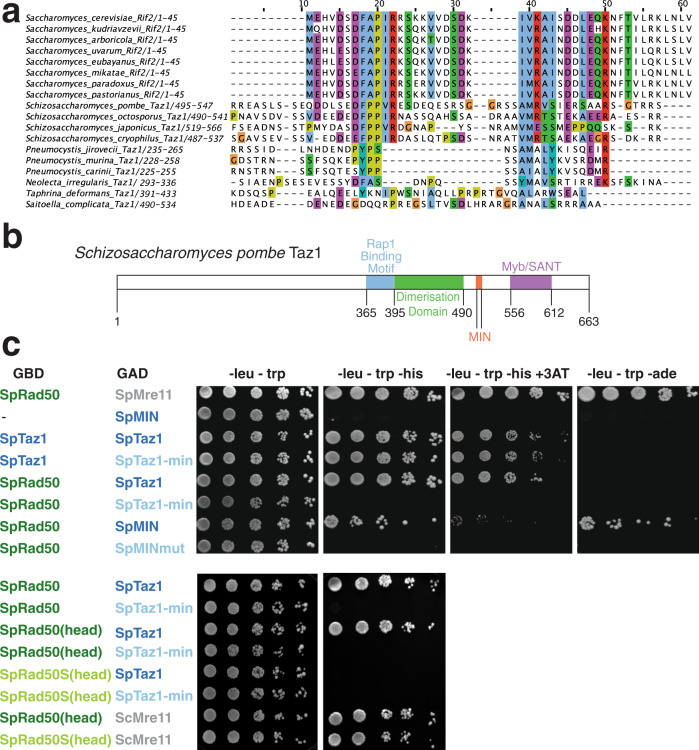
A MIN motif in fission yeast Taz1 binds the Rad50 N terminus. **a** Sequences of putative Taz1 proteins from the Taphrinomycotina were aligned using Clustal Omega with the N-terminal region of Rif2 from *Saccharomyces* species. **b** Diagram of the domain structure of *Schizosaccharomyces pombe* Taz1. Domains previously identified in SpTaz1^76^ are indicated for the C-terminal half of the protein. The MIN motif is in orange. **c** Yeast two-hybrid assays of the *S. pombe* fusion proteins are indicated. Plates are minimal medium lacking the nutrients indicated on top (and one instance containing 3 mM 3-aminotriazole). Five-fold serial dilutions were spotted left to right and incubated for 3–4 days at 30 °C. Source data are provided as a Source Data file.

The fission yeast *Schizosaccharomyces pombe* is a well-studied model system for telomere biology. When we searched for the MIN motif in *S. pombe*, we found a match in Taz1. In *S. pombe* the telomeric complex does not rely on Rap1 for binding to telomeric DNA repeats, but utilizes instead the DNA-binding protein Taz1. Remarkably, this putative MIN motif was conserved in all four *Schizosaccharomyces* species whose genome has been sequenced (Fig. 7a). Although several residues from the extended consensus derived from Rif2 and Orc4 sequences, particularly in its N-terminal region, were absent, the four invariant residues in the core motif at positions +1, +2, +5 and +6 (Fig. 1c) were present. The highly conserved proline at +4 was also present, and a stringent requirement seems to operate in *Schizosaccharomyces* at the otherwise relaxed position +3. Intriguingly, the motif is located in the C-terminal part of the protein, in a small region of little sequence conservation outside the MIN motif itself and of no known function, which is sandwiched between the Myb/SANT DNA-binding domain and a homodimerization domain (Fig. 7b). In *S. pombe* strains carrying the same F to E and P to N mutations (*taz1-min*) equivalent to those characterized in *S. cerevisiae*, we did not observe strong changes in telomere length: this is not surprising as telomere length is not dependent on MRN-Tel1 in this species^64^. Similarly, we failed to observe strong defects in telomere protection in this mutant. In fission yeast, deprotected telomeres undergo fusions in a manner dependent on MRN when cells are kept in a prolonged G1 arrest. Strains carrying *taz1-min* showed no obvious effect on cell viability. This result is consistent with the major role played by Rap1 in the suppression of NHEJ in *S. pombe*^65^.

Because telomeres possess redundant pathways to disarm the various branches of the DDR, we hypothesise that the MIN motif in *S. pombe* might act redundantly with other telomere factors in dealing with the threat posed by untimely activation of MRN. We decided to address the possibility that Taz1 might target MRN by assessing the ability of the Taz1 MIN to interact with Rad50. Using yeast two-hybrid assays, an interaction between Taz1 and Rad50 was readily observed (Fig. 7c). Crucially, the interaction was abolished when the Taz1-min (F511E, P513N) protein was used, while this protein retained, as expected, the ability to homodimerize, and is therefore functional. Finally, binding to Rad50 was retained when only the region of Taz1 between the dimerization and DNA-binding domains was included (amino acids 489–558); again, the introduction of the F511E, P513N double mutation abolished the ability of the 70 amino acids spanning the MIN motif to interact with Rad50. These results show that the MIN motif is required for the interaction of Taz1 with Rad50 and that a small part of the Taz1 protein overlapping the MIN motif is sufficient for this interaction, and thus mimic and extend the interaction data obtained with Rif2 and Rad50 in budding yeast. Strikingly, a K81I mutation in *S. pombe* Rad50, equivalent to the same change in the *S. cerevisiae* protein (Rad50S), abolished the interaction between Rad50 and Taz1, strongly suggesting a conserved mode of interaction between Rif2/Taz1 and Rad50 (Fig. 7c, bottom panels). These results predict that Taz1 interacts with Rad50 in fission yeast cells. It remains to be seen whether the Taz1 motif has a similar role to Rif2 in modulating the activity of MRN. Because Taz1 is evolutionarily unrelated to Rif2 or Orc4, we suggest that the MIN motif has appeared independently in the Schizosaccharomycetes. We also note that the motif is not present in other Taz1 proteins from the Taphrinomycotina (Fig. 7a), indicating that the motif appeared in this clade after the appearance of Taz1, which is of uncertain origin but might be linked to the transcription factor Tbf1.

## Discussion

We presented evidence that several yeast species have evolved a protein motif that has the remarkable ability to dismantle the multiple functionalities of the MRN/X complex at telomeres. The available evidence suggests that the MIN motif achieves this by promoting non-productive ATP hydrolysis by the MRN/X complex, and thus abolishing the functions of MRN/X that depend on productive ATP hydrolysis. Our biochemical data suggest that the MIN motif appears to hijack a regulatory step normally, leading to the activation of the endonuclease activity of MRN/X to instead disable it. Our data show that every phenotype characteristic of the *rif2*-null allele that we have tested is reproduced by mutating MIN within the protein. Perhaps, even more remarkably, every MRX activity that we have tested, save for exonucleolytic activity of Mre11, is disabled by MIN. Further, MIN is not just a simple binding interface tasked with the job of delivering a larger disabling activity of some sort; rather, as little as 34–40 amino acids encompassing MIN are shown to be sufficient both to bind Rad50 and to suppress DNA repair and NHEJ in vivo by MRN, and as little as 60 can inhibit Tel1 activation in vivo at telomeres^38^. How MIN achieves this remains to be determined, but is likely to shed important light on regulatory conformational transitions in MRN^39^. The surprising finding that Rif2 and Sae2 share a binding interface in Rad50 also points to a potential additional regulatory role for MIN in affecting Sae2 activity on MRX.

In budding yeast, the action of MRX-Tel1 is not only a potentially dangerous trigger for DDR activation but is also required for telomerase action, which occurs preferentially at shorter telomeres. Mre11 appears to facilitate telomerase recruitment specifically to the leading strand telomere, possibly by promoting its resection^66^. Remarkably, Rif2 marks the shorter telomeres for preferential telomerase action^67^, so it is possible that maintaining a threshold of telomere-bound MIN motifs is required for efficient MRN inhibition. This mechanism could potentially help limit the spurious action of MIN at non-telomeric sites, where its nucleation might not reach the required threshold^23^. Similar considerations might apply to fission yeast telomeres, but the exact role for the Taz1 MIN motif remains to be determined. We note that human cells actively recruit MRN to telomeres^68,69^ and that the role of ATM in telomerase activation has recently been documented^12,13^. In addition, MRN can have a protective role at mammalian telomeres, by preventing telomere fusions^70,71^. Thus, it is possible that the fine-tuning of MRN action in higher eukaryotes requires strategies similar to those allowed by the deployment of the MIN motif in yeast. Interestingly, *rad50S* alleles were found to lead to increased telomere fusions and genome instability in mice, raising the possibility that similar mechanisms to control MRN action at telomeres might exist in higher eukaryotes^72^.

## Methods

### Strains and plasmids

All *S. cerevisiae* (*Sc*) strains used in this study were derivatives of W303 (*leu2-3,112 trp1-1 can1-100 ura3-1 ade2-1 his3-11,15). Sc* and *K. lactis* (*Kl*) strains were grown in YPAD-rich medium or synthetic complete (SC) minimal medium (2% w/v glucose, 0.67% w/v Yeast Nitrogen Base, 0.2% w/v SC dropout mix, 2% w/v agar, pH 6.0). YNB and SC mixes were from United States Biological. For transformations, the LiAc protocol was followed, with heat shock at 42 °C for 45 min. A list of strains is provided as Supplementary Table 1.

The *rif2-min* allele was generated by transformation of *Sc* strains with plasmid pAB1703, which contains a small genomic *RIF2* region into which the F8E P10N mutations were engineered: the plasmids were linearised with *Bsr*GI before transformation, transformants were selected on SC-ura medium and then counter-selected on 5FOA-containing medium to identify recombination events that popped out the inserted plasmid from the *RIF2* locus. Transformants that retained the mutations were screened by PCR using the silent *Bgl*II introduced alongside the mutations. A similar approach was used for the *orc4-min* allele in *K. lactis* using plasmid pAB2049 linearized with *Eco*RI.

For overexpression of the Rif2 MIN motif in *Sc*, two plasmids were used, containing the first 34 amino acids of Rif2, either in wild-type or F8E P10N form, N terminally fused to a GFP-binding protein fragment, expressed from a galactose-inducible promoter (pAB2081 and pAB2082, respectively). The plasmids were linearized with *Bst*XI before being transformed into *Sc* strains. Integration of the plasmids at the *HIS3* locus was confirmed by PCR.

The *rad50S* allele was introduced in the required genetic background using pNKY349 (pAB2301) cut with *Bam*HI–*Eco*RI. Correct transformants were screened by PCR and MMS sensitivity. Transformants that bore the correct plasmid integration at the *RAD50* locus but retained the wild-type *RAD50* allele after recombination of the construct, were also selected and used in the CPT and MMS sensitivity experiments to ensure an equal assortment of auxotrophic markers in spotting assays in *RAD50* wild-type vs mutant comparisons. For the NHEJ experiments, a variant of the pNKY349 plasmid where the URA4 marker was swapped with HIS3 was constructed, pScRad50-K81I-Hi (pAB2304), and used in the same way.

### Southern blotting analysis

*Sc* or *Kl* genomic DNA was prepared from 10 ml log-phase cultures in YPAD-rich media by vortexing cell pellets with 0.3 g glass beads in 0.4 ml 2% Triton X, 1% sodium dodecyl sulfate (SDS), 100 mM NaCl, 10 mM Tris-HCl pH 8.0, 1 mM ethylenediaminetetraacetic acid (EDTA) pH 8.0, followed by phenol:chloroform:isoamyl alcohol (25:24:1) extraction, ethanol precipitation and RNase A digestion. For telomere length analysis, 1 μg of genomic DNA was digested with *Xho*I (*S. cerevisiae*) or *Eco*RI (*Kl*) overnight and gel electrophoresis was carried out on 1% agarose gels. Depurination was carried out by soaking the gel in 125 mM HCl for 10 min, followed by denaturation in 0.5 M NaOH 1.5 M NaCl for 30 min. The overnight transfer was in 20× saline sodium citrate (SSC) using nylon membranes (Roche). After ultraviolet crosslinking, membranes were incubated at 60 °C (*Sc*) or 50 °C (*Kl*) in 5× SSC, 5% dextran sulfate, 0.2% I-Block (Thermo Fisher T2015) and 0.1% SDS. DNA probes were briefly denatured before being added, and hybridization was carried out overnight. Washes were at room temperature with buffers pre-heated at 60 °C (*Sc*) or 50 °C (*Kl*) twice in 1× SSC 0.1% SDS and then twice in 0.5× SSC 0.1% SDS. Detection was by incubation for 1 h at room temperature (RT) in 100 mM Tris pH 7.55, 150 mM NaCl, 1% skimmed milk, plus 1 μl of alkaline phosphatase-tagged anti-fluorescein F(ab) (Roche) (1:125,000), followed by four washes at RT in 100 mM Tris pH 7.55, 150 mM NaCl, and detection with CDP-Star detection agent. Imaging was on a LAS4000 instrument. Probes were made either by PCR of a Y′ fragment followed by random priming (*Sc*) or by terminal transferase extension of a 25-mer telomeric oligonucleotide (*Kl*), both in the presence of Fl-dUTP. Southern data were collected using an Image Quant LAS4000 instrument (GE), images were processed using Adobe Photoshop and lanes were analysed using Image Gauge v4.1 (Fuji).

### Single-cell checkpoint arrest analysis

Strains were grown overnight in 5 ml of YPLG (acid lactic and glycerol) medium. The following morning, cells were diluted into 5 ml of the same medium to 3 × 10^5^ cells/ml and incubated at 30 °C. After 2 h of incubation, 1 ml of 20% galactose was added in order to induce the HO endonuclease gene and the strains were incubated for a further 2 h. After that, the cells were plated down in the central line of a YPAD plate, kept previously at room temperature. Then, small-budded cells were dissected into a grid by a micromanipulator for analysis. After 1 h from the beginning of the dissection, cells were checked every 30 min for the second round of budding in order to record the cell cycle restart. YPAD plates were preserved at 30 °C in an incubator between observations. After the growth of the colonies at 30 °C, the following day, the central line of the plate was removed and it was incubated for a further one day. The next morning, the strains were replicated in Sc-Lys plate and incubated for 2 days at 30 °C. In the end, Lys^+^ colonies were excluded from the dataset because the LYS2 gene is located on the telomere-proximal side of the HO site and only cells Lys^**−**^ that had been subjected to a DNA break were scored. The average restart time for each construct was estimated using Kaplan–Meier survival analysis in GraphPad.

### NHEJ assays

Strains were grown on a solid minimal medium containing glucose and lacking uracil and tryptophan, and then grown to saturation in the same medium overnight, before being diluted and plated on the same plates (to calculate plating efficiency, used for normalisation), and also on minimal plates lacking tryptophan and containing glucose^33^.

### Yeast two-hybrid analysis

Yeast two-hybrid assays were performed by co-transforming GBD and GAD (Gal4-activating domain) plasmids (in the pGBKT7 and pGADT7 vectors) in various combinations in the PJ69-4A budding yeast strain (*MATa trp1-901 leu2-3,112 ura3-52 his3-200 gal4 gal80 LYS2::GAL1-HIS3 GAL2-ADE2 met2::GAL7-lacZ*). Transformants were screened for interaction by spotting 5-fold dilutions on minimal plates lacking tryptophan and leucine for selection of GBD and GAD plasmids, respectively. Interactions were assessed by quantification of the expression of the *HIS3* and *ADE2* markers by plating on minimal medium lacking, in addition, histidine or adenine. The stringency of the *HIS3* reporter was also increased by the addition of 3 mM 3-aminotriazole. The plates were then incubated at 30 °C for 3 (SC-leu-trp) or 4 (selective plates) days, imaged with a Syngene InGenius instrument and processed with Photoshop.

### Recombinant protein purification

*Saccharomyces cerevisiae* Mre11-Rad50-Xrs2 (MRX, containing Mre11-his, Xrs2-FLAG and untagged Rad50), Mre11-Rad50 (MR, containing Mre11-his and untagged Rad50), Mre11 (his-tagged at the C terminus), Rad50 (FLAG-tagged at the C terminus), Rad50S (K81I, FLAG-tagged at the C terminus) and Xrs2 (FLAG-tagged at the C terminus) proteins were prepared after expression in *Spodoptera frugiperda* 9 (*Sf*9) cells by affinity chromatography^28,51,52,73^. Recombinant phosphorylated Sae2, containing an MBP tag at the N terminus and a His-tag at the C terminus, was expressed in *Sf*9 cells and purified by sequential affinity chromatography steps using amylose resin (New England Biolabs), followed by the cleavage of the MBP tag with PreScission protease (12 μg of protease per 100 μg MBP-Sae2-His). Finally, pSae2 was purified using NiNTA agarose (Qiagen)^52^.

Wild-type Rif2 and Rif2-min variants were expressed using pFB-MBP-Rif2-his or pFB-MBP-Rif2-min-his vectors, respectively. The *Sf*9 cell pellets (from 1000 ml culture) were resuspended in 4 volumes of lysis buffer (50 mM Tris-HCl pH 7.5, 1 mM dithiothreitol, 1 mM EDTA, 1:400 Sigma protease inhibitory cocktail P8340, 1 mM phenylmethylsulfonyl fluoride, 30 μg/ml leupeptin) and incubated for 20 min at 4 °C. One-half volume of 50% glycerol and 6.5% volume 5 M NaCl solutions were then added, and the sample was incubated for 30 min while stirring. The cell extract was centrifuged (48,000 × *g* for 30 min) to obtain the soluble extract. The extract was bound to 6 ml amylose resin (New England Biolabs) for 1 h batchwise. The resin was washed with wash buffer (50 mM Tris-HCl pH 7.5, 5 mM 2-mercaptoethanol, 1 M NaCl, 10% glycerol, 1 mM phenylmethylsulfonyl fluoride), and protein was eluted with elution buffer (50 mM Tris-HCl pH 7.5, 5 mM 2-mercaptoethanol, 300 mM NaCl, 10% glycerol, 1 mM phenylmethylsulfonyl fluoride, 10 mM maltose). The protein concentration was estimated by the Bradford method and PreScission protease (1:5, w/w) was added, and the sample was incubated for 2 h at 4 °C. Then, 10 mM imidazole (final concentration) was added to the sample and the solution was bound to 0.5 ml NiNTA resin (Qiagen). The resin was washed with NTA buffer A1 (50 mM Tris-HCl pH 7.5, 5 mM 2-mercaptoethanol, 1 M NaCl, 10% glycerol, 0.5 mM phenylmethylsulfonyl fluoride, 58 mM imidazole) and NTA wash buffer A2 (50 mM Tris-HCl pH 7.5, 5 mM 2-mercaptoethanol, 150 mM NaCl, 10% glycerol, 58 mM imidazole) and eluted with NTA buffer B (50 mM Tris-HCl pH 7.5, 5 mM 2-mercaptoethanol, 150 mM NaCl, 10% glycerol, 300 mM imidazole). The eluate was dialysed into 50 mM Tris-HCl pH 7.5, 5 mM 2-mercaptoethanol, 150 mM NaCl, 10% glycerol, aliquoted, frozen in liquid nitrogen and stored at −80 °C. The purification yielded ~1 ml of 10 μM Rif2 (his-tagged at the C terminus). The Rif2-min variant was prepared in the same way.

### ATPase assays

The reaction buffer contained 25 mM Tris-acetate pH 7.5, 5 mM magnesium acetate, 1 mM dithiothreitol, 0.25 mg/ml bovine serum albumin (BSA) (New England Biolabs), 150 μM cold ATP, 1 nM labelled γ-^32^P-ATP, 50 nM DNA substrate (in molecules, annealed oligonucleotides 210-GTAAGTGCCGCGGTGCGGGTGCCAGGGCGTGCCCTTGGGCTCCCCGGGCGCGTACTCCACCTCATGCATC and 211-GATGCATGAGGTGGAGTACGCGCCCGGGGAGCCCAAGGGCACGCCCTGGCACCCGCACCGCGGCACTTAC) and respective recombinant proteins. The reactions were incubated for 4 h at 30 °C. The reactions were stopped with EDTA (50 mM final concentration). The substrate and hydrolysed ATP were separated by thin-layer chromatography using 0.3 M lithium chloride and 0.3 M formic acid (mixed 1:1) as the mobile phase. The plates were then dried and exposed to storage phosphor screens and a signal was detected using Typhoon imager (GE Healthcare).

### Nuclease assays

The 50-bp-long DNA substrate was prepared by annealing oligonucleotides PC1253C (AACGTCATAGACGATTACATTGCTAGGACATCTTTGCCCACGTTGACCCA) and PC1253B (TGGGTCAACGTGGGCAAAGATGTCCTAGCAATGTAATCGTCTATGACGTT) with 3′-terminal biotin^28^. Upon labelling with ^32^P, annealing and purification, the substrate was bound to streptavidin to create a protein block. The buffer for the nuclease assays contained 25 mM Tris-acetate pH 7.5, 1 mM dithiothreitol, 0.25 mg/ml BSA (New England Biolabs), 1 nM DNA substrate (in molecules), 5 mM magnesium acetate, 1 mM manganese acetate, 1 mM ATP, 80 Us/ml pyruvate kinase, 1 mM phosphoenolpyruvate and the recombinant proteins, as indicated. The assays were incubated for 30 min at 4 °C, and the reaction products were analysed by electrophoresis under denaturing conditions and autoradiography.

### Electrophoretic mobility shift assays

To monitor DNA binding, assays (15 μl) were carried out in 25 mM Tris-acetate pH 7.5, 1 mM dithiothreitol, 5 mM magnesium acetate, 1 mM manganese acetate, 0.25 mg/ml BSA (New England Biolabs), 1 mM phosphoenolpyruvate, 1 mM ATP, 80 U/ml pyruvate kinase and 1 nM ^32^P-labelled dsDNA substrate (in molecules, annealed oligonucleotides PC1253C and PC1253B). Afterwards, recombinant proteins were added, and the reactions were incubated at 30 °C for 30 min. Afterwards, the samples were mixed with 4 μl 50% glycerol with bromophenol blue, and separated by electrophoresis in 6% polyacrylamide in TAE buffer. Gels were dried and developed by autoradiography.

### Pull-down assays

The *Sf*9 cell lysate containing ~5 μg of MBP-Rif2 or MBP-Rif2-min (see recombinant protein preparation) was incubated for 1 h at 4 °C with amylose resin (New England Biolabs, 50 μl per reaction). The resin with the immobilized MBP-Rif2 variants was washed five times with 1 ml wash buffer (50 mM Tris-HCl pH 7.5, 200 mM NaCl, 0.2% NP40, 2 mM EDTA, Sigma protease inhibitory cocktail P8340 1:1000), using 500 × *g* for 2 min centrifugation each time to pellet the resin. Afterwards, 1 μg of Mre11, 500 ng of Rad50, or 1 μg of Xrs2 were added to the resin and incubated together for 1 h in the same wash buffer. Afterwards, the resin was washed four times with 1 ml wash buffer to remove unbound proteins. Bound proteins were then eluted with 100 μl wash buffer supplemented with 20 mM maltose. PreScission protease was then added (0.5 μg) to cleave the MBP tag from MBP-Rif2 or MBP-Rif2-min. The proteins in the eluate were separated by electrophoresis and detected by Western blotting. The antibodies used were: anti-yRad50 (Thermo Scientific, PA5-32176, 1:1000); anti-FLAG (Sigma, F3165, 1:1000); anti-His (MBL, D291-3, 1:5000). Pull-down assays with the Rad50-K81I variant were carried out as above with the following modifications: the resin with the immobilized MBP-Rif2 variants was first washed four times with 1 ml wash buffer containing 1 M NaCl (50 mM Tris-HCl pH 7.5, 1 M NaCl, 0.2% NP40, 2 mM EDTA, Sigma protease inhibitory cocktail P8340 1:1000), and then once with the same buffer but containing 150 mM NaCl. Afterwards, 750 ng of recombinant Rad50 or Rad50-K81I were added to the resin and incubated for 1 h at 4 °C in wash buffer containing 150 mM NaCl. The same buffer was then used in washes to remove the unbound proteins, as described above.

### Statistics and reproducibility

All DNA and protein gels were repeated at least twice with similar results. Yeast two-hybrid assays were conducted with pools of transformants. Measurements were taken from distinct samples.

### Reporting summary

Further information on research design is available in the Nature Research Reporting Summary linked to this article.

## Supplementary information

Supplementary Information

Reporting Summary

## Data Availability

The data supporting the findings of this study are available from the corresponding authors upon reasonable request. Uncropped images of gels, blots and spot assays are provided with this paper. Source data are provided with this paper.

## Source data

Source Data
