# Supplementary material for: Inhibition of MRN activity by a telomere protein motif
Source: Nat Commun. 2021 Jun 22;12:3856. doi: 10.1038/s41467-021-24047-2 (PMC8219681; doi:10.1038/s41467-021-24047-2)
Supplement: Supplementary file 1 — Supplementary Information [file 41467_2021_24047_MOESM1_ESM.pdf]

Inhibition of MRN activity by a telomere protein motif

Supppementary Table 1.

List of strains used in this study. All S. cereviasiae strains are in the W303 background. K. lactis strains are indicated by ‘KL’ in the number.

YAB0	*MATa*	Lab collection.
YAB1	*MATα*	Lab collection.
YAB594	*MATa bar1-D lys2::pGAL-ISceI ISceI::URA3::ISceI rad50S:URA3 [pAB2124]*	This study.
YAB595	*MATa bar1-D lys2::pGAL-ISceI ISceI::URA3::ISceI::5UASGal4 [pAB2123]*	This study.
YAB717	*MATa bar1-D lys2::pGAL-ISceI ISceI::URA3::ISceI::5UASGal4 [pAB2124]*	This study.
YAB734	*MATa bar1-D lys2::pGAL-ISceI ISceI::URA3::ISceI [pAB2123]*	This study.
YAB781	*MATa bar1-D lys2::pGAL-ISceI ISceI::URA3::ISceI [pAB2124]*	This study.
YAB1517	*MATa ade2-1 his3-200 leu2-3,112 trp1-901 ura3-52 gal4-Δ gal80-Δ MET2::GAL7-lacZ LYS2::GAL1-HIS3 GAL2-ADE2*	Lab collection.
YAB1759	*MATa rif2-min*	This study.
YAB1760	*MATa rif2-min*	This study.
YAB1761	*MATa bar1-D lys2::pGAL-ISceI ISceI::URA3::ISceI::5UASGal4*	Marcand, S., et al., *Genes Dev* 22, 1153-1158 (2008).
YAB1764	*MATa bar1-D lys2::pGAL-ISceI ISceI::URA3::ISceI::5UASGal4 rif2::HPH*	Marcand, S., et al., *Genes Dev* 22, 1153-1158 (2008).
YAB1765	*MATa bar1-D lys2::pGAL-ISceI ISceI::URA3::ISceI*	Marcand, S., et al., *Genes Dev* 22, 1153-1158 (2008).
YAB1778KL	*Mata lysA1 trp1 leu2 metA1 uraA1 mre11::kanMX*	Stefan U. Åström.
YAB1789	*MATa bar1-D lys2::pGAL-ISceI ISceI::URA3::ISceI::5UASGal4 rif2-min*	This study.
YAB1984	*MATa bar1-D lys2::pGAL-ISceI ISceI::URA3::ISceI::5UASGal4 tel1::kanMX*	This study.
YAB1990	*MATα::loxP ade2::loxP leu2::pG-HO-L2 lys2::loxP mnt2::LYS2 mnt2::TG80-HO-CA80-ADE2*	Ribeyre, C. & Shore, D., *Nat Struct Mol Biol* 19, 307-313 (2012).
YAB1991	*MATα::loxP ade2::loxP leu2::pG-HO-L2 lys2::loxP mnt2::LYS2 mnt2::TG80-HO-CA80-ADE2 rif1::NatMX4*	Ribeyre, C. & Shore, D., *Nat Struct Mol Biol* 19, 307-313 (2012).
YAB1992	*MATα::loxP ade2::loxP leu2::pG-HO-L2 lys2::loxP mnt2::LYS2 mnt2::TG80-HO-CA80-ADE2 rif2::KanMX4*	Ribeyre, C. & Shore, D., *Nat Struct Mol Biol* 19, 307-313 (2012).
YAB1993	*MATα::loxP ade2::loxP leu2::pG-HO-L2 lys2::loxP mnt2::LYS2 mnt2::TG80-HO-CA80-ADE2 rif1::NatMX4 rif2::KanMX4*	Ribeyre, C. & Shore, D., *Nat Struct Mol Biol* 19, 307-313 (2012).
YAB2004KL	*MATα ade2-202 ura3*	Dudy Tzfati.
YAB2029	*MATa xrs2-664*	Shima, et al., *Genetics* 170, 71-85 (2005).

YAB2035	*MATα::loxP ade2::loxP leu2::pG-HO-L2 lys2::loxP mnt2::LYS2 mnt2::TG80-HO-CA80-ADE2 rif2-min*	This study.
YAB2037	*MATα::loxP ade2::loxP leu2::pG-HO-L2 lys2::loxP mnt2::LYS2 mnt2::TG80-HO-CA80-ADE2 rif1::NatMX4 rif2-min*	This study.
YAB2038	*MATα::loxP ade2::loxP leu2::pG-HO-L2 lys2::loxP mnt2::LYS2 mnt2::TG80-HO-CA80-ADE2 rif1::NatMX4 rif2-min*	This study.
YAB2039	*MATα rif2-min*	This study.
YAB2047	*MATa xrs2::TRP1*	This study.
YAB2052	*MATa rif2-min xrs2-664*	This study.
YAB2053	*MATα rif2-min xrs2-664*	This study.
YAB2054	*MATa rif2-min xrs2-664*	This study.
YAB2057	*MATa rif2::ADE2*	This study.
YAB2058	*MATa rif2::ADE2*	This study.
YAB2060	*MATα rif2::ADE2*	This study.
YAB2065	*MATa rif2::ADE2 xrs2::TRP1*	This study.
YAB2067	*MATa rif2::ADE2 xrs2::TRP1*	This study.
YAB2106	*MATa tel1::TRP1*	This study.
YAB2110	*MATa rif2::ADE2 tel1::TRP1*	This study.
YAB2111	*MATa rif2::ADE2 tel1::TRP1*	This study.
YAB2112	*MATa rif2-min xrs2::TRP1*	This study.
YAB2114	*MATa rif2-min tel1::TRP1*	This study.
YAB2115	*MATa rif2-min tel1::TRP1*	This study.
YAB2116	*MATα rif2-min xrs2::TRP1*	This study.
YAB2143KL	*trp1 leu2 met? ura3 rif1::URA3*	This study.
YAB2144KL	*lysA1 leu2 met? ura3 rif1::URA3*	This study.
YAB2145KL	*leu2 met? ura3 mre11::kanMX orc4-min rif1::URA3*	This study.

YAB2155KL	*lysA1 leu2 met? ura3 orc4-min rif1::URA3*	This study.
YAB2157KL	*MATa ade2-Δ ura3*	Jürgen J. Heinisch.
YAB2158KL	*MATα ade2-Δ ura3*	Jürgen J. Heinisch.
YAB2171KL	*lysA1 met? ura3 mre11::kanMX*	Carter, et al., *Genetics* 175, 1035-1045 (2007).
YAB2174KL	*MATα ura3 orc4-min*	This study.
YAB2175KL	*MATa ura3 orc4-min*	This study.
YAB2176KL	*MATa ura3 orc4-min*	This study.
YAB2177KL	*MATα ura3 orc4-min*	This study.
YAB2188KL	*MATα ura3 orc4-min rif1::URA3*	This study.
YAB2189	*MATa RAD50:URA3*	This study.
YAB2190	*MATa his3::Pgal-MIN RAD50:URA3*	This study.
YAB2191	*MATa his3::Pgal-MINmut RAD50:URA3*	This study.
YAB2192	*MATa rad50S:URA3*	This study.
YAB2193KL	*lysA1 met? ura3 mre11::kanMX orc4-min*	This study.
YAB2194	*MATa his3::Pgal-MIN rad50S:URA3*	This study.
YAB2195	*MATa his3::Pgal-MIN rad50S:URA3*	This study.
YAB2196	*MATa his3::Pgal-MINmut rad50S:URA3*	This study.
YAB2197KL	*leu2 met? trp1 ura3 mre11::kanMX rif1::URA3*	This study.
YAB2199KL	*leu2 met? ura3 mre11::kanMX orc4-min rif1::URA3*	This study.
YAB2200	*MATa bar1-D lys2::pGAL-ISceI ISceI::URA3::ISceI rad50S:URA3 [pAB2123]*	This study.
YAB2201	*MATa rif2::ADE2*	This study.
YAB2204	*MATa bar1-D lys2::pGAL-ISceI ISceI::URA3::ISceI::5UASGal4 rad50S:URA3 [pAB2124]*	This study.
YAB2205	*MATa bar1-D lys2::pGAL-ISceI ISceI::URA3::ISceI::5UASGal4 rad50S:URA3 [pAB2123]*	This study.

YAB2284	*MATα rif2-min tel1::TRP1 rad50S:URA3*	This study.
YAB2285	*MATa rif2::ADE2 tel1::TRP1 rad50S:URA3*	This study.
YAB2286	*MATα tel1::TRP1 rad50S:URA3*	This study.
YAB2287	*MATα rif2-min rad50S:URA3*	This study.
YAB2288	*MATa rif2-min rad50S:URA3*	This study.
YAB2289	*MATa rif2-min rad50S:URA3*	This study.
YAB2290	*MATα rif2::ADE2 rad50S:URA3*	This study.
YAB2291	*MATa rif2::ADE2 rad50S:URA3*	This study.
YAB2292	*MATa rif2::ADE2 rad50S:URA3*	This study.
YAB2293	*MATα rad50S:URA3*	This study.
YAB2294	*MATα rad50S:URA3*	This study.
YAB2295	*MATa his3::Pgal-MIN*	This study.
YAB2296	*MATa his3::Pgal-MINmut*	This study.
YAB2297	*MATα his3::Pgal-MIN*	This study.
YAB2298	*MATα his3::Pgal-MINmut*	This study.
YAB2310KL	*MATα ade2-Δ leu2 ura3 rif1::URA3*	This study.
YAB2311KL	*MATα ade2-Δ leu2 ura3 rif1::URA3*	This study.
YAB2349KL	*MATa ura3 orc4-min rif1::URA3*	This study.
YAB2351	*MATa rad50S:URA3*	This study.
YAB2352KL	*MATa ura3 orc4-min rif1::URA3*	This study.
YAB2353KL	*MATa ura3 orc4-min rif1::URA3*	This study.
YAB2355KL	*MATα ura3 orc4-min rif1::URA3*	This study.
