# Supplementary material for: Inhibition of MRN activity by a telomere protein motif
Source: Nat Commun. 2021 Jun 22;12:3856. doi: 10.1038/s41467-021-24047-2 (PMC8219681; doi:10.1038/s41467-021-24047-2)
Supplement: Supplementary file 2 — Reporting Summary [file 41467_2021_24047_MOESM2_ESM.pdf]

nature research

Corresponding author(s): Alessandro Bianchi

Last updated by author(s): May 5, 2021

Reporting Summary

Nature Research wishes to improve the reproducibility of the work that we publish. This form provides structure for consistency and transparency in reporting. For further information on Nature Research policies, see Authors & Referees and the Editorial Policy Checklist.

Statistics

For all statistical analyses, confirm that the following items are present in the figure legend, table legend, main text, or Methods section.

n/a	Confirmed	
☐	☒	The exact sample size (*n*) for each experimental group/condition, given as a discrete number and unit of measurement
☐	☒	A statement on whether measurements were taken from distinct samples or whether the same sample was measured repeatedly
☐	☒	The statistical test(s) used AND whether they are one- or two-sided<br>*Only common tests should be described solely by name; describe more complex techniques in the Methods section.*
☒	☐	A description of all covariates tested
☒	☐	A description of any assumptions or corrections, such as tests of normality and adjustment for multiple comparisons
☐	☒	A full description of the statistical parameters including central tendency (e.g. means) or other basic estimates (e.g. regression coefficient) AND variation (e.g. standard deviation) or associated estimates of uncertainty (e.g. confidence intervals)
☐	☒	For null hypothesis testing, the test statistic (e.g. *F*, *t*, *r*) with confidence intervals, effect sizes, degrees of freedom and *P* value noted<br>*Give P values as exact values whenever suitable.*
☒	☐	For Bayesian analysis, information on the choice of priors and Markov chain Monte Carlo settings
☒	☐	For hierarchical and complex designs, identification of the appropriate level for tests and full reporting of outcomes
☒	☐	Estimates of effect sizes (e.g. Cohen's *d*, Pearson's *r*), indicating how they were calculated

Our web collection on statistics for biologists contains articles on many of the points above.

Software and code

Policy information about availability of computer code

Data collection	Protein sequence alignments were done using Clustal Omega at the EBI website interface (https://www.ebi.ac.uk/Tools/msa/clustalo/). Southern data were collected using an Image Quant LAS4000 instrument (GE), and lanes analysed using Image Gauge v4.1 (Fuji) . Yeast plates were imaged using a Syngene InGenius instrument. Biochemical data were collected using commercial software, available as a package with the respective instrument: Typhoon FLA 9500 capture software (GE Healthcare, Version 1.0, Build 1.0.0.185), and Fusion FX7 capture software (Vilber Imaging, Version FX7 Edge 18.06 -SN).
Data analysis	Analysis/display of sequence alignments was carried out using Jailview 2.11.1.4. Analysis/presentation of Southern and yeast spotting experiments was achieved using Adobe Photoshop. Image J (NIH, Version 1.52q) and ImageQuant (GE Healthcare, TL 7.0) were used for the analysis of biochemical data. Graphs and numerical data (including statistics/error bars) were analyzed and plotted by Excel (Microsoft Excel for Mac v.16.40) and Prism (GraphPad, Version 8.2.1).

For manuscripts utilizing custom algorithms or software that are central to the research but not yet described in published literature, software must be made available to editors/reviewers. We strongly encourage code deposition in a community repository (e.g. GitHub). See the Nature Research guidelines for submitting code & software for further information.

Data

Policy information about availability of data

All manuscripts must include a data availability statement. This statement should provide the following information, where applicable:

- Accession codes, unique identifiers, or web links for publicly available datasets
- A list of figures that have associated raw data
- A description of any restrictions on data availability

All relevant data generated or analyzed during this study are included in this manuscript. All relevant data are available from the authors upon reasonable request. Source data are provided with this paper, including uncropped images of gels, blots and spot assays.

Field-specific reporting

Please select the one below that is the best fit for your research. If you are not sure, read the appropriate sections before making your selection.

☒ Life sciences ☐ Behavioural & social sciences ☐ Ecological, evolutionary & environmental sciences

For a reference copy of the document with all sections, see nature.com/documents/nr-reporting-summary-flat.pdf

Life sciences study design

All studies must disclose on these points even when the disclosure is negative.

Sample size	Sample size (or number of repeats) was not predetermined. Sample size was based on what is common in the field, and what was practical to do. For budding yeast and K. lactis telomere length analysis, multiple (2 or 3) independently derived strains of the most important genotypes were analysed. A similar approach was carried out for the viability assays showed in Fig.2, where each genotype was generate and tested in duplicate. For yeast two-hybrids pools of mutliple transformants were tested. For the genetic experiments of Figure 3 the standard practice of assessing the data from a minimum of three independent experiments was adopted. Only for the rif1-Δ strains in Fig.3a two independent experiments were obtained. For the NHEJ assays in Fig.3b, for the various genotypes shown left to right, the number of independent cultures analysed was 3, 3, 6, 6, 3, 3, 3, 3, 3, 10, 9.
Data exclusions	For the genetic experiments, no data were excluded, except for obvious technical failures, such as contaminated plates, etc. For the biochemical experiments, in general, no data were excluded unless there was a valid reason to do so, e.g. experiments with failed positive controls indicating technical problems, or when loading control indicated unequal loading that invalidated the analysis or other technical issues (broken gels, collapsed wells in gels etc.).
Replication	For telomere length analysis, for all the most relevant genotypes, multiple independently derived strains (2-4) were analysed, and analysis was carried out in 2-3 gels. Three independent strains were analysed for each genotype in Figure 5e, and the experiment was repeated with similar results. Yeast two-hybrid analysis (Figures 5b and 7c) was carried out 2-3 times with independent isolates with similar results. Genotoxicity experiments (Figure 5d) were carried out multiple times (and at varying drug concentrations) with similar qualitative results. For the biochemical experiments, either 5 (nuclease assays) or two repeats (ATPase assays and DNA binding) were carried out, with similar results. All attempts at replication, save for technical failure, were successful.
Randomization	Randomization is not relevant to the experiments performed nor was the use of covariates, as they could not be applied to the types of experiments carried out in this study.
Blinding	Blinding was not carried out as measurements were either objectively quantified or simply visually presented and hence were not prone to subjective judgment by the experimentalists. Also, the way the data needed to be organised for presentation (loading order etc) prevented blinding. In addition, blinding would have been largely impossible or at least highly impractical to execute as many experiments involved a single experimentalist.

Reporting for specific materials, systems and methods

We require information from authors about some types of materials, experimental systems and methods used in many studies. Here, indicate whether each material, system or method listed is relevant to your study. If you are not sure if a list item applies to your research, read the appropriate section before selecting a response.

Materials & experimental systems

n/a	Involved in the study	
☐	☒	Antibodies
☒	☐	Eukaryotic cell lines
☒	☐	Palaeontology
☒	☐	Animals and other organisms
☒	☐	Human research participants
☒	☐	Clinical data

Methods

n/a	Involved in the study	
☒	☐	ChIP-seq
☒	☐	Flow cytometry
☒	☐	MRI-based neuroimaging

Antibodies

Antibodies used	Antibodies used were: anti yRad50 mThermo Scientific PA5-32176 (1:1000), anti FLAG Sigma F3165 (1:1000) , anti-His MBL D291-3 (1:5000). For Southerns alkaline phosphatase tagged anti-fluorescein F(ab) (Roche 11 426 338 910) (1:125,000) was used.
Validation	The yRad50 antibody was validated against yeast lysates by the manufacturer (https://www.thermofisher.com/antibody/product/RAD50-Antibody-Polyclonal/PA5-32176) and against purified recombinant protein by us. The other antibodies (anti-FLAG, -His, and -fluorescin antibodies) are well-characterised standard commercial anitbodies of general use.
